# Genome Sequencing of the SARS-CoV-2 Delta (B.1.617.2) Variant of Concern Detected in Bangladesh

**DOI:** 10.1128/MRA.00849-21

**Published:** 2021-12-02

**Authors:** Tanjina Akhtar Banu, M. Murshed Hasan Sarkar, Shahina Akter, Barna Goswami, Iffat Jahan, Eshrar Osman, Mohammad Samir Uzzaman, M. Ahashan Habib, Abu Sayeed Mohammad Mahmud, Mohammad Mohi Uddin, Tasnim Nafisa, M. Maruf Ahmed Molla, Mahmuda Yeasmin, Arifa Akram, M. Salim Khan

**Affiliations:** a Bangladesh Council of Scientific & Industrial Research (BCSIR), Dhaka, Bangladesh; b SciTech Consulting and Solutions, Dhaka, Bangladesh; c National Institute of Laboratory Medicine and Referral Center, Dhaka, Bangladesh; KU Leuven

## Abstract

We report the near-complete genome sequence and phylogenetic analysis of a severe acute respiratory syndrome coronavirus 2 (SARS-CoV-2) Delta variant (B.1.617.2) strain. This variant is associated with increased transmission and immune evasion.

## ANNOUNCEMENT

Severe acute respiratory syndrome coronavirus 2 (SARS-CoV-2) is the causative agent of coronavirus disease 2019 (COVID-19) and belongs to the family *Coronaviridae* and the genus *Betacoronavirus*. More than 1 year after its emergence, it is associated with record numbers of cases and deaths (1). SARS-CoV-2 variants of concern (VOC) B.1.1.7 (501Y.V1/Alpha), B.1.351 (501Y.V2/Beta), B.1.1.28.1 (501Y.V3/Gamma), and B.1.617.2 (20A/S:478K/Delta) were observed during the sudden surge in COVID-19 cases in the UK, South Africa, Brazil, and India, respectively, with subsequent spread across the world (2, 3). The B.1.617.2 variant was first detected India in December 2020 (4), and on 11 May 2021, the World Health Organization classified it as VOC Delta, due to its increased transmissibility (5). The BCSIR conducted the sequencing with ethical approval from the National Institute of Laboratory Medicine and Referral Center, Dhaka, Bangladesh (NILMRC) (reference number 224125200).

**FIG 1:**
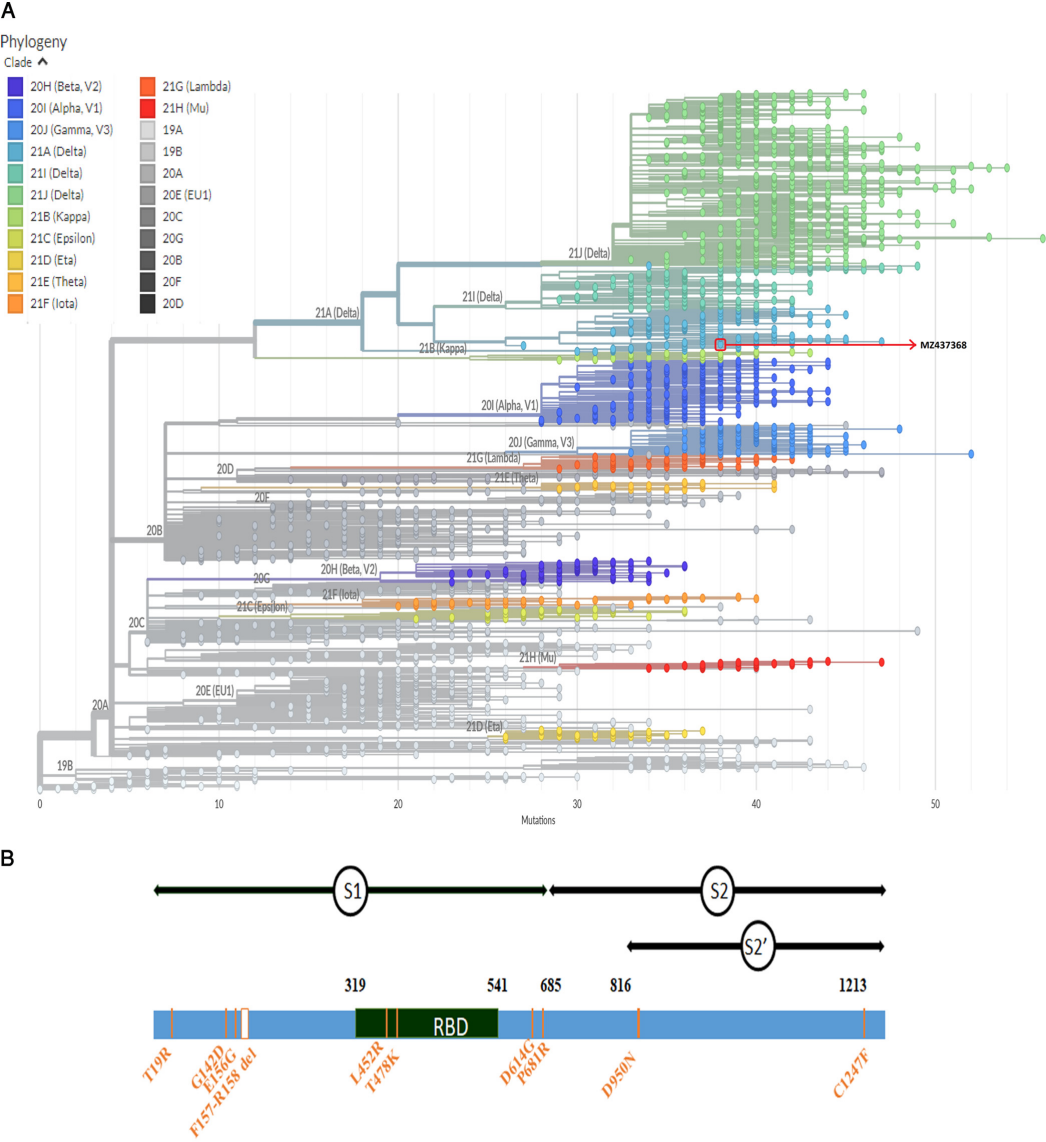
(A) Phylogenetic tree of the genome submitted under GenBank accession number MZ437368 from the Bangladeshi sample. The FASTA file was uploaded in Nextclade (https://clades.nextstrain.org/results), and a phylogenetic tree was created using MZ437368 to represent the Delta lineage (B.1.617.2/20A/S:478K) in Bangladesh. (B) Schematic overview of various mutations in the spike protein region of the SARS-CoV-2 Delta variant (B.1.617.2) from Bangladesh compared with the reference strain Wuhan-Hu-1 (NC_045512.2).

In this study, we report the near-complete genome sequence of a SARS-CoV-2 Delta variant strain in Bangladesh. A patient visited the outdoor unit of Brahmanbaria General Hospital, Brahmanbaria, with a fever, throat pain, respiratory distress, severe weakness, etc. An oropharyngeal swab specimen from the patient tested positive for COVID-19 at the NILMRC on 18 May 2021 (*C_T_* = 11.9; open reading frames [ORF] = 12.91; S = 12.31) using a commercial one-step real-time COVID-19 PCR kit (Sansure Biotech, Inc., Changsha, China). At the BCSIR, viral RNA was extracted using the ReliaPrep viral total nucleic acid purification kit (Promega, USA). A sequencing library was prepared using the Illumina RNA prep with enrichment (L) tagmentation kit according to the manufacturer’s instructions. The library was sequenced using the MiniSeq sequencing system with an output of 2 × 74-bp paired-end reads. Sequencing generated 10,942,096 reads, of which 10,518,454 unique reads were found after excluding duplicate marked reads using BaseSpace DRAGEN RNA Pathogen Detection software version 3.5.1 with default settings. Using BaseSpace DRAGEN RNA Pathogen Detection software, a FASTA file was generated from the FASTQ files, resulting in a near-complete genome sequence of the Bangladeshi SARS-CoV-2 strain (BCSIR_NILMRC_757). The near-complete genome sequence was 29,860 bp long and showed an average GC content of 38.0%. The variant analysis was performed on the EzBioCloud server (6), which uses strain Wuhan-Hu-1 (GenBank accession number NC_045512.2) as the reference genome to determine the variants. The Pangolin lineage was determined and the phylogenetic analysis performed using the default parameters of Nextclade version 1.7.1 (https://clades.nextstrain.org/tree) (7). Pangolin lineage analysis confirmed that the virus belongs to lineage B.1.617.2 (Fig. 1A).

We found a total of 11 mutations in the S protein of the Delta variant (Fig. 1B). Nine mutations are located within the S1 subunit, five of which are present in the N-terminal domain (T19R, G142D, and E156G and two deletions, F157-, R158-), two in the receptor-binding domain (RBD) (L452R and T478K), and two between the RBD and the S1/S2 border (D614G and P681R). Two additional mutations are located within the S2′ subunit (D950N and C1247F) (Fig. 1B). L452R, T478K, and P681R are three key mutations in the spike protein; it has been suggested that they are responsible for making the strain more transmissible and infectious (8).

It has been confirmed that this patient did not report any recent travel history or known contact with recent travelers, so it is possible that this infection resulted from local community transmission of the SARS-CoV-2 Delta variant (B.1.617.2) in Bangladesh in May 2021.

### Data availability.

The sequence has been deposited at GenBank under accession number MZ437368. The raw sequence reads have been deposited at the NCBI Sequence Read Archive (SRA) under BioProject accession number PRJNA741723 and SRA accession number SRP336330.
